# Larval Settlement: The Role of Surface Topography for Sessile Coral Reef Invertebrates

**DOI:** 10.1371/journal.pone.0117675

**Published:** 2015-02-11

**Authors:** Steve Whalan, Muhammad A. Abdul Wahab, Susanne Sprungala, Andrew J. Poole, Rocky de Nys

**Affiliations:** 1 Marine Ecology Research Centre, School of Environment, Science and Engineering, Southern Cross University, PO Box 157, Lismore, 2480, New South Wales, Australia; 2 MACRO—the Centre for Macroalgal Resources and Biotechnology, James Cook University, Townsville, 4811, Queensland, Australia; 3 Australian Institute of Marine Science, PMB 3 Townsville, Queensland, 4810, Australia; 4 AIMS@JCU, James Cook University, Townsville, 4811, Queensland, Australia; 5 ARC Centre for Excellence for Coral Reef Studies, James Cook University, Townsville, 4811, Queensland, Australia; 6 College of Public Health, Medical and Veterinary Sciences, Department of Molecular Sciences, James Cook University, Townsville, 4811, Queensland, Australia; 7 CSIRO Manufacturing Flagship, Pigdons Road, Waurn Ponds, 3216, Victoria, Australia; University of New South Wales, AUSTRALIA

## Abstract

For sessile marine invertebrates with complex life cycles, habitat choice is directed by the larval phase. Defining which habitat-linked cues are implicated in sessile invertebrate larval settlement has largely concentrated on chemical cues which are thought to signal optimal habitat. There has been less effort establishing physical settlement cues, including the role of surface microtopography. This laboratory based study tested whether surface microtopography alone (without chemical cues) plays an important contributing role in the settlement of larvae of coral reef sessile invertebrates. We measured settlement to tiles, engineered with surface microtopography (holes) that closely matched the sizes (width) of larvae of a range of corals and sponges, in addition to surfaces with holes that were markedly larger than larvae. Larvae from two species of scleractinian corals (*Acropora millepora* and *Ctenactis crassa*) and three species of coral reef sponges (*Luffariella variabilis*, *Carteriospongia foliascens* and *Ircinia* sp.,) were used in experiments. *L. variabilis*, *A. millepora* and *C. crassa* showed markedly higher settlement to surface microtopography that closely matched their larval width. *C. foliascens* and *Ircinia* sp., showed no specificity to surface microtopography, settling just as often to microtopography as to flat surfaces. The findings of this study question the sole reliance on chemical based larval settlement cues, previously established for some coral and sponge species, and demonstrate that specific physical cues (surface complexity) can also play an important role in larval settlement of coral reef sessile invertebrates.

## Introduction

Understanding processes that contribute to distribution patterns of organisms is a cornerstone of ecology. Indeed, for spatially structured populations, dispersal behaviour and habitat selection underpins distribution patterns [1]. Moreover, when habitat selectivity is linked to dispersive individuals, habitat choices can have implications for Darwinian fitness, particularly in complex or heterogeneous landscapes [2,3]. Importantly, the selection of suboptimal sites to settle can be maladaptive in comparison to optimal habitats where opportunities for success and fitness are highest [2,4].

Habitat selection can revolve around complex behaviours with some taxa demonstrating defined and innate behaviours that optimise selection [5,6]. This is often displayed for breeding [7], with marine turtles returning to natal beaches to lay eggs [8], brood parasitism in birds [9,10], and insects using chemical cues to oviposit eggs in habitats that minimise predatory risks, or optimise food resources for emerging larvae [11] being among the many examples identifying the use of behaviour to provide optimal habitats for offspring.

Marine sessile invertebrates commonly exhibit complex life cycles with adults occupying a sessile form while the larval or propagule phase is planktonic. Population distributions can therefore be moderated by a complex range of processes involving both benthic (adult) and planktonic (larval) cycles [12,13]. The roles of adult reproduction, larval dispersal and settlement, and juvenile post-settlement success in defining adult distributions and demography are difficult to disentangle [14]. Nevertheless, adult distributions are primarily directed by the larval phase, thereby highlighting the importance of larval behaviour in habitat selectivity during dispersal [15] and once larvae engage settlement sites [16].

Chemical (environmental) cues that signal habitat and illicit larval settlement are a common denominator for a wide range of sessile marine taxa with settlement initiated in response to conspecifics [17], host organisms [18] and microorganism biofilms [19–21]. Two of the most interesting chemical cues implicated in the settlement of coral reef invertebrates involve crustose coralline algae (CCA) and microbial biofilms.

CCA are a conspicuous calcifying algae found on shallow (photic zone) coral reefs [22], where several species of CCA illicit settlement (metamorphosis) responses in a range of scleractinian corals [23–25], soft corals [26] and sponges [16]. While coral larval settlement in response to CCA, and associated microbial biofilms [27,28], has been a focus for coral reef larval ecologists in recent decades the molecules that induce settlement are largely unidentified [29,30] preventing it from being universally useful. Moreover, settlement in response to CCA is observed to be highly variable, and different species of CCA elicit different settlement responses [31]. Chemical cues associated with microorganism biofilms also contribute to larval settlement for a wide range of sessile invertebrates, including sponges and corals [20,21,32]. Consistent with settlement responses of coral larvae to CCA, settlement to biofilms can also be variable for some species [33]. Larval settlement is complex and dynamic, but our incomplete understanding of settlement processes on coral reefs highlight the need to raise questions about the sole importance of chemical cues in larval settlement, particularly at the expense of physical cues such as surface topography.

Compared to chemical cues, the effect of physical microtopography on larval settlement is less well studied. Surface structure, at topographical scales that approximate larval sizes (i.e. < 1 mm), is important for some temperate invertebrate larvae, such as barnacles [34–36]. Surfaces structured with crevices of several millimetres have been used in coral and sponge recruitment studies [37–41] but these studies demonstrated settlement to surface topography that were considerably larger than larval/propagule dimensions. There is considerable knowledge on the role of microtopography in the fields of marine biofouling and bio-mimicry [42,43] with a predominant focus on determining methods to impede larval settlement. One of the key findings from this research is that settlement and adhesion of marine invertebrate larvae can be influenced by the complexity of surface microtopography, a concept developed through “Attachment Point Theory” [44]. If microtopography provides surfaces with texture that closely match larval dimensions, then settlement is increased by increasing the available points of attachment for larvae or propagules [45,46]. Conversely, surfaces that are smaller than larval dimensions minimise the number of attachment points and often reduce settlement success [44] and the strength of adhesion [47,48].

The broad objective of this study was to explore the premise that there are cues, beyond those of a chemical nature, that induce larvae to settle, and that physical surface structure is important. To undertake this we test the hypothesis that surface microtopography contributes to larval settlement, and that the relationship between the size of surface microtopography and the size of larvae (Attachment Point Theory) is important for the settlement of larvae of corals and sponges.

## Materials and Methods

### Test organisms

Scleractinian (hard) corals often dominate shallow coral reef communities. Sponges can also form an important component of the coral reef ecosystems. Accordingly, we selected the corals *Acropora millepora* and *Ctenactis crassa*, and the sponges *Carteriospongia foliascens*, *Ircinia* sp., and *Luffariella variabilis*. *A*. *millepora* and *L*. *variabilis* were important inclusions because both species have previously shown strong preferences for settlement to chemical based cues. *A*. *millepora* larvae settle to CCA cues [24] and *L*. *variabilis* to conspecific cues [17].

### Coral larvae rearing

*A*. *millepora* and *C*. *crassa* are locally abundant corals found throughout the shallow waters of the Great Barrier Reef. *A*. *millepora* is hermaphroditic and *C*. *crassa* is gonochoric. Both species are broadcast-spawning corals with non-feeding larvae that lack zooxanthellae when released.

Larvae were cultured from three colonies of *A*. *millepora* and one female and four male specimens of *C*. *crassa*. All corals were collected from the fringing reefs of Orpheus Island, central Great Barrier Reef, two days prior to the full moon in November 2012, transported to Orpheus Island Research Station (OIRS), and maintained in flow through aquaria until spawning occurred in December 2012. Gametes from all *A*. *millepora* colonies were collected, combined, and left for 2 h to fertilize. Eggs of the female *C*. *crassa* polyp were collected on the 4^th^ of December 2012, combined with sperm from 3 male polyps, and left for fertilization for 1.5 hours. After fertilization, developing embryos were transferred to 500-L aquaria containing 1 μm filtered seawater (FSW). The aquaria were continuously supplied with flow through FSW, at a flow rate of approximately 2 L min^-1^. The aquaria were maintained for eight days in a temperature controlled room at 27C with a 12:12 h light: dark cycle. The majority of larvae showed settlement behaviour (cork-screw like swimming towards bottom of aquaria) at six days after spawning and were then used in settlement assays.

### Sponge larval collection

*Luffariella variabilis*, *Carteriospongia foliascens* and *Ircinia* sp. are brooding dictyoceratid sponges that are locally abundant within the Great Barrier Reef [49,50]. These three species dribble spawn parenchymella larvae daily for several weeks during the Austral summer and are competent to settle within three days of release [17,51,52] (Whalan unpublished data). Up to 10 individual sponges from each species were collected from the fringing reefs of Orpheus Island in December 2012 and transported to OIRS where they were maintained in flow through aquaria. Larvae were collected by placing mesh traps over sponges following Whalan et al [33], and immediately used in larval experiments.

### Larval sizes

Larval size is important in interpreting the effect that specific sizes of microtopography have on larval settlement choices. Therefore, 50 larvae from each of the five species were collected at spawning (sponges), or just prior to use in settlement assays (corals), and fixed in 2.5% glutaraldehyde to enable measurements of larval width and length.

### Experimental settlement surfaces and design

**Fig 1:**
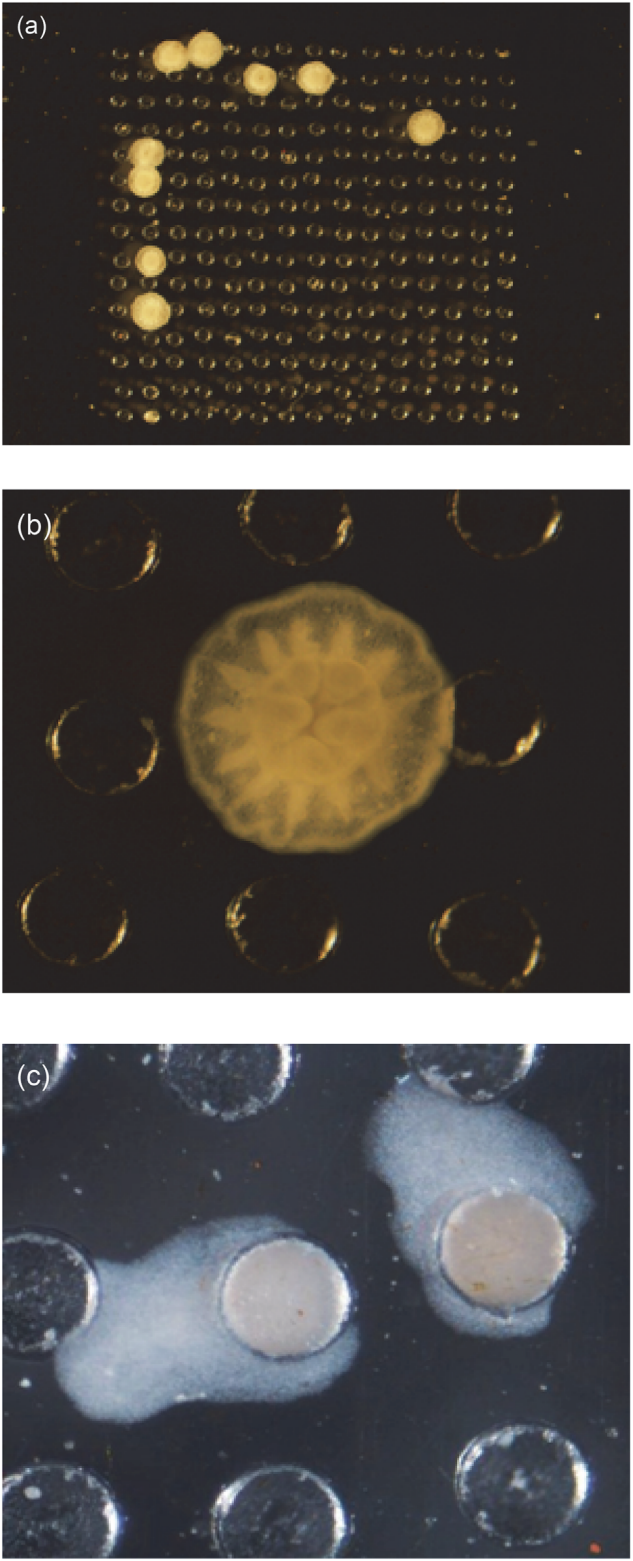
a-c: Photographs of coral and sponge recruits following metamorphosis within holes, and subsequent growth beyond holes. (a) *Acropora millepora* recruits and (b) detail of development of radial septa and a central oral disc of an *A*. *millepora* juvenile. (c). *Luffariella variabilis* recruits.

Artificial surfaces engineered with a range of different sized surface microtopography were used to test the effect of surface microtopography on larval settlement. Surfaces comprised 50 mm × 50 mm × 5 mm polycarbonate tiles (Makrolon UV, Bayer Material Science). Surface treatments were prepared by drilling 200 μm deep holes at 400 μm, 700 μm or 1000 μm diameters using a Hartford VMC 1020 numerically controlled mill with Heidenhain controller at a drill speed of 8000 rpm. Surfaces were designed with a 15 × 15 grid of holes (see Fig. 1a for an example of 400 μm treatments). The distances between each hole therefore provided an inter-hole, flat surface. Control surfaces included flat tile surfaces without microtopography, although it is noted that the crevice between the tile and vial also presents a form of microtopography, both within controls and all other treatments.

Larval settlement assays for each species were undertaken by pipetting 20 larvae into 20 mL glass scintillation vials holding 15 mL of 10 μm filtered sea water (FSW), placing the artificial settlement tile over the opening of the scintillation vial, and carefully inverting the vial so that larvae were in direct contact with the test surface. Vials were then secured to surfaces with rubber bands and maintained in temperature controlled rooms with 12:12 photo period cycles. A total of 10 replicate tiles for each of the surface sizes were used for each species. Larval metamorphosis was scored every six hours using an inverted microscope. Larval metamorphosis was scored in both holes and on the flat surface between holes for all surface treatments, and the flat surface of controls. While we acknowledge there are differences between the terms of “settlement” and “metamorphosis” [21] for clarity we use the term “settlement” as a descriptor of the process of larval settlement through to larval metamorphosis from this point on.

### Analysis

The effect of surface microtopography on settlement was established with several different analyses. Firstly, to explore the effect of larval size and settlement to sizes of microtopography (among all five species) data was analysed using Principal Coordinate Analysis (Primer E). Secondly, we analysed the effect of surfaces on settlement, treating surface microtopography as a fixed factor, using one way Analysis of Variance (ANOVA). Repeated measures ANOVA was used to analyse the effect of surface microtopography on settlement over time, with time as the within factor variable and surface treatment as the (fixed) between factor variable. Finally, to establish if settlement was occurring randomly to holes or non-holes, for each surface microtopography treatment, we tested the data against a goodness of fit binomial probability model (settlement to a hole versus non hole) based on the respective surface areas of holes and no holes within each tile. Because each tile treatment comprised holes at different diameters the ratio of surface area between “holes” and “no holes” differed between surface treatments. This difference was accounted for in the analysis by including the ratio of the surface area taken up by holes to no holes in the probability model as follows: 400 μm—0.25:0.75; 700 μm—0.2:0.8; 1000 μm—0.55:0.45; for holes to no holes respectively. Unless otherwise stated all data are reported as means ± one standard error.

## Results

### Larval behaviour and sizes

Larvae from species of both corals and sponges exhibited an ovoid shape at release and during their planktonic phase. Larval behaviours were similar amongst species. During the settlement phase larvae actively explored surfaces and periodically made contact with a surface for several seconds, often spending extended durations in holes prior to settlement. In general larvae exhibited a behaviour whereby they rotated on their posterior-anterior pole during settlement and prior to metamorphosis. Metamorphosis included a transition from a typical larval form to a flattened disk-like morphology (sponges) or distinctive polyp form (corals) (Fig. 1 a-c).

Given the preference for larvae to commence settlement by rotating on their aboral-oral axis the relevant metric of larval size in relation to the holes defining the surface microtopography is larval width. Mean widths of larvae ranged from 273 μm for the smallest species (*C*. *crassa*) to 504 μm for *C*. *foliascens* (Table 1). The range of surface microtopography constituted surfaces with holes having diameters of 400 μm, 700 μm and 1000 μm. The 400 μm treatment closely resembled larval width for *A*. *millepora*, *L*. *variabilis* and *Ircinia* sp., with larger sized holes (700 μm and 1000 μm) being 200–400 μm larger than the larval widths of all five species.

**Table 1:** Summary of mean larval dimensions (n = 50).

Species	Length (μm) ± SE	Width (μm) ± SE
A. millepora	891.65 ± 13.52	434.33 ± 6.35
C. crassa	364.93 ± 2.98	272.30 ± 2.43
L. variabilis	629.25 ± 8.17	391.76 ± 5.78
C. foliascens	864.39 ± 15.68	504.63 ± 9.92
*Ircinia* sp.	756.77 ± 3.27	447.47 ± 4.52

### Settlement to surface microtopography

**Fig 2:**
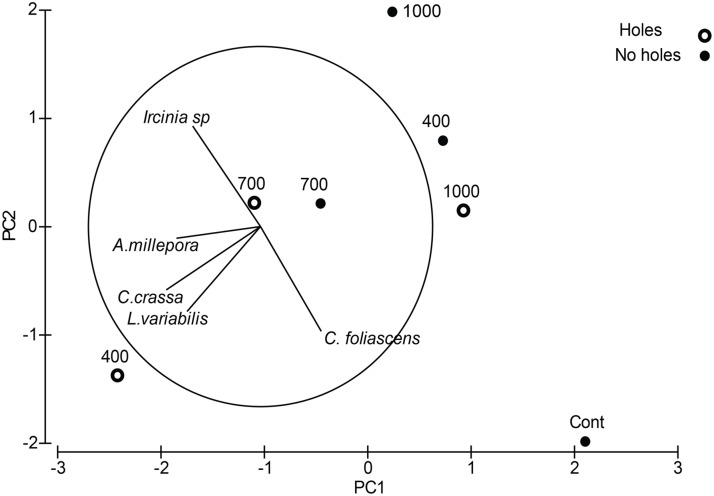
Ordination following Principal Components Analysis of larval metamorphosis for the corals *Acropora millepora*, *Ctenactis crassa*, and the sponges *Luffariella variabilis*, *Carteriospongia foliascens* and *Ircinia* sp. to a range of surface microtopography containing circular pits outlined in a grid fashion engineered into artificial polycarbonate tiles. Settlement choices include “holes” or the “flat surface between holes”. Settlement to flat surface controls (Cont) is also included. Total variation in the two principal components is equivalent to 78.9% (PC1 = 43.8% and PC2 = 35.1%).

*A*. *millepora*, *C*. *crassa* and *L*. *variabilis* larvae showed clear responses of selective settlement to 400 μm holes, whilst the other remaining sponge species, *Ircinia* sp., and *C*. *foliascens*, showed less specific responses to surface microtopography. This broad trend is clear within the PCA ordination with *C*. *crassa*, *L*. *variabilis*, and *A*. *millepora* grouping near 400 μm holes (Fig. 2). In contrast, *C*. *foliascens* and *Ircinia* sp., settlement occurred across the spectrum of surface microtopographies.

**Fig 3:**
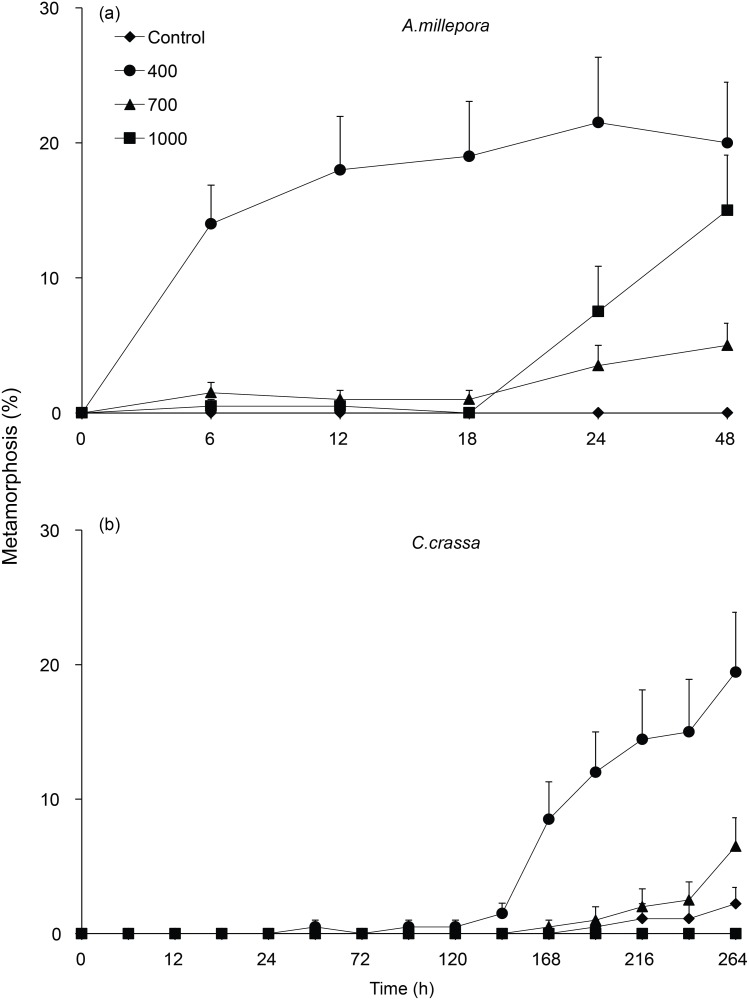
a-b. Larval metamorphosis (mean % + 1 SE) for the corals, *Acropora millepora* and *Ctenactis crassa*, in response to surface microtopography treatments of smooth controls, or surfaces with 400 μm, 700 μm and 1000 μm diameter holes. Total larval age at the termination of the experiment, including the culture period, is 9 days for *A*. *millepora* and 17 days for *C*. *crassa*.

**Fig 4:**
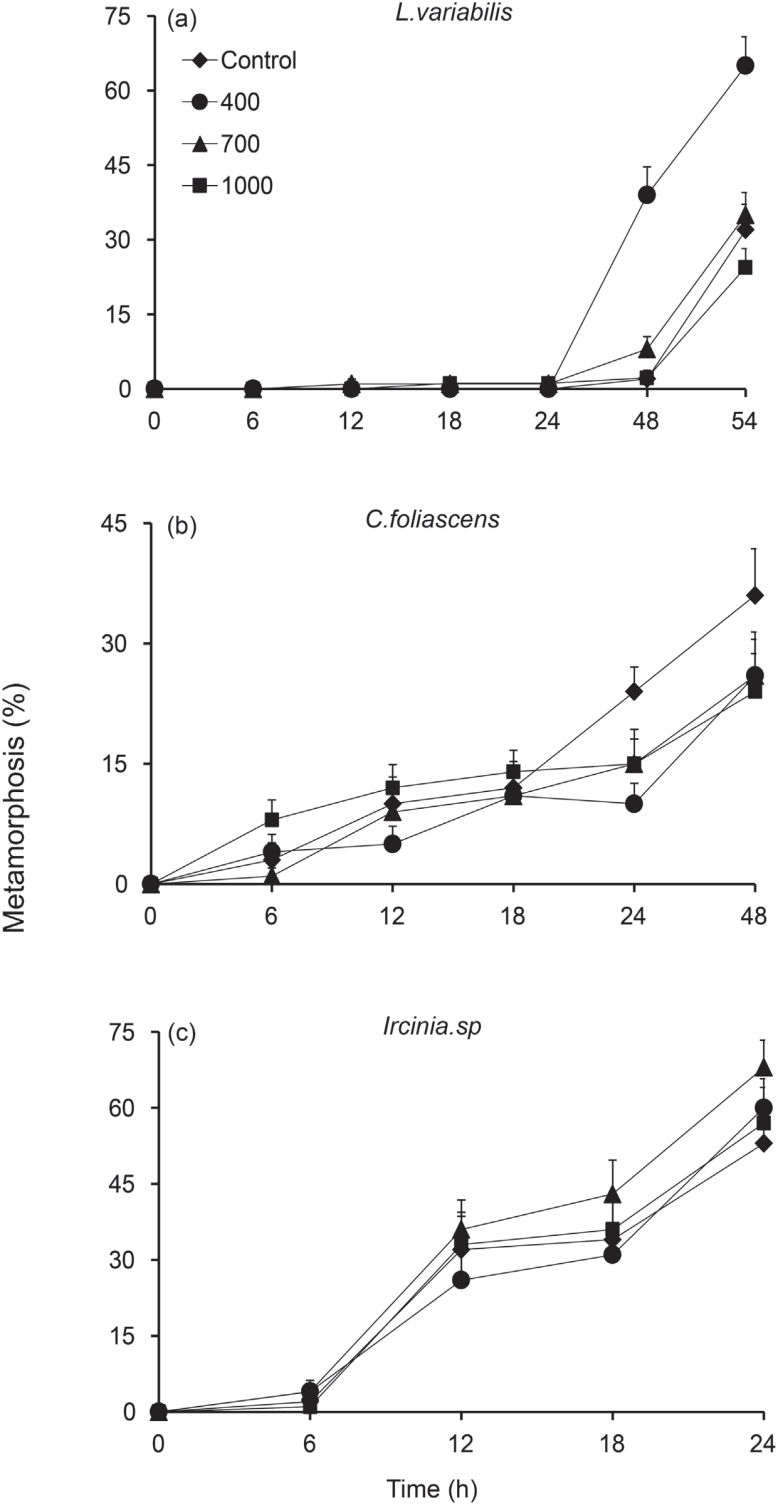
a-c. Larval metamorphosis (mean % + 1 SE) for the sponges, *Luffariella variabilis*, *Carteriospongia foliascens*, and *Ircinia* sp. in response to surface microtopography treatments of smooth controls, or surfaces with 400 μm, 700 μm and 1000 μm diameter holes. Settlement occurred within 3 days of spawning for all three species.

There were significant effects of surface microtopography on larval settlement for the two coral species, *A*. *millepora* and *C*. *crassa*, and the sponge *L*. *variabilis* at the completion point of each experiment (Fig. 3 a-b; Fig. 4a, Table 2). For *A*. *millepora*, settlement in 400 μm treatments reached a mean of 20 ± 4.49%, and this sized treatment also corresponded with the mean larval width of this species (Table 1). There was no settlement of *A*. *millepora* in controls. *A*. *millepora* settlement in the 400 μm treatments was significantly higher than larval settlement in 700 μm (5 ± 1.63%) and 1000 μm (15 ± 4.08%) treatments, both of which showed consistent levels of settlement (Fig. 3a). There was no settlement of *C*. *crassa* to 1000 μm treatments and larval metamorphosis to both 400 μm (19.44 ± 4.4%) and 700 μm (6.52 ± 1.1%) treatments was significantly higher compared to control surfaces (2.22 ± 1.22%); noting that the mean larval width of *C*. *crassa*, at 272 μm, was smaller than all hole treatments tested (Fig. 3b; Table 1). *L*. *variabilis* had increased settlement in 400 μm treatments and this size topography closely matched mean larval width (Table 1). *L*. *variabilis* settlement reached 65 ± 5.82% in 400 μm compared to 35 ± 4.53%, 24.44 ± 3.76 and 32 ± 5.12% in 700 μm, 1000 μm and controls respectively (Fig. 4a).

**Table 2:** Summary statistics for PERMANOVA analysis of endpoint metamorphosis to surface treatments.

Species	Source	df	MS	(Pseudo) F	p
A. millepora	Size	3	12193.00	17.719	0.001
	Residuals	27	668.10		
C. crassa	Size	3	12478.00	13.195	0.001
	Residuals	36	941.90		
L. variabilis	Size	3	3205.40	6.564	0.001
	Residuals	35	488.27		
*Ircinia* sp.	Size	3	376.61	10.991	0.341
	Residuals	36	342.64		
C. foliascens	Size	3	444.60	0.399	0.899
	Residuals	36	1113.40		

For the sponges *Ircinia* sp. and *C*. *foliascens* there was no effect of surface treatment on larval settlement. Both these species showed consistent levels of settlement between holes and no-holes (Table 2; Fig. 4 b-c) irrespective of surface treatments.

Settlement also occurred on the walls of vials, or at the vial-tile interface for all three sponge species. Settlement to vial walls was highly inconsistent among species, and surface treatments, and in many instances occurred in a single replicate. These results preclude reliable statistical analysis beyond reporting a summary of cases where settlement occurred (Table 3). No larval settlement was recorded on the walls of the vials for the two coral species.

**Table 3:** Summary of larval metamorphosis (%) that occurred on the walls of vials, or at the tile wall/interface of glass vials.

Species	400	700	1000	Control
A. millepora	0	0	0	0
C. crassa	0	0	0	0
*Ircinia* sp.	20 (n = 1)*	20 (n = 1)*	20 (n = 1)*	17.5 (n = 3)
L. variabilis	10 (n = 1)*	6.5 (n = 3)	0	11.5 (n = 7)
C. foliascens	6.5 (n = 8)	9.5 (n = 8)	8.5 (n = 6)	17 (n = 10)

Numbers are averages with replicates noted in brackets. Note several results are based on only one replicate, (noted with an asterisk), and therefore not indicative of average metamorphosis.

### Settlement to surfaces over time

*A*. *millepora*, *L*. *variabilis*, *C*. *foliascens* and *Ircinia* sp. larvae completed settlement within three days, and *C*. *crassa* settlement occurred over an 11 day period (Fig. 3 a-b, Fig. 4 b-c). *A*. *millepora*, *C*. *crassa* and *L*. *variabilis* larvae all showed significant interactions between time and treatment effects (*A*. *millepora—Pseudo F*_*12*, *177*_ *= * 2.74, p < 0.01; *C*. *crassa—Pseudo F*_*39*,*495*_ *= * 10.06, p < 0.01; *L*. *variabilis—Pseudo F*_*5*, *210*_ = 3.73, p < 0.01). Settlement for the remaining two sponges, *C*. *foliascens* and *Ircinia* sp., was affected by time only (*C*. *foliascens—Pseudo F*_*4*, *186*_ = 14.46, p < 0.01; *Ircinia* sp., *Pseudo F*_*3*, *144*_ = 73.94, p < 0.01), confirming, in part, the results at the completion point of settlement assays (Fig. 4 b-c).

### Settlement: hole versus no hole

**Fig 5:**
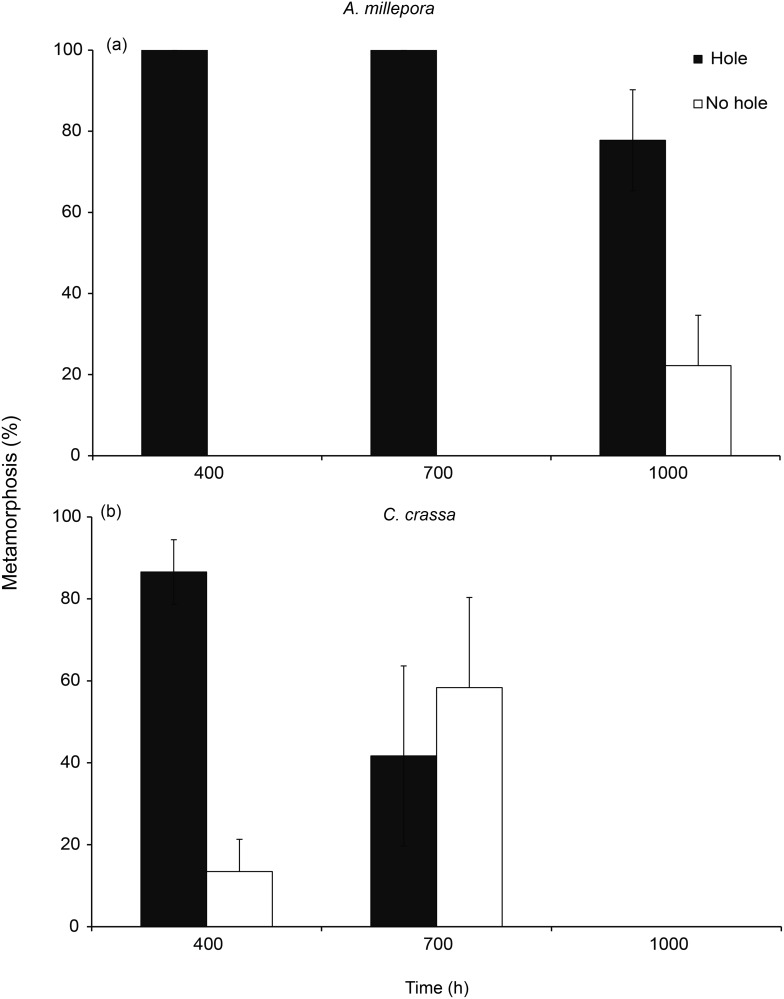
a-b. Larval metamorphosis (mean % + 1 SE) for the corals, *Acropora millepora* and *Ctenactis crassa*, that occurred in holes or no holes (flat inter-hole surfaces) for surface microtopography treatments of 400 μm, 700 μm and 1000 μm diameter holes. Total larval age at the termination of the experiment, including the culture period, is 9 days for *A*. *millepora* and 17 days for *C*. *crassa*.

**Fig 6:**
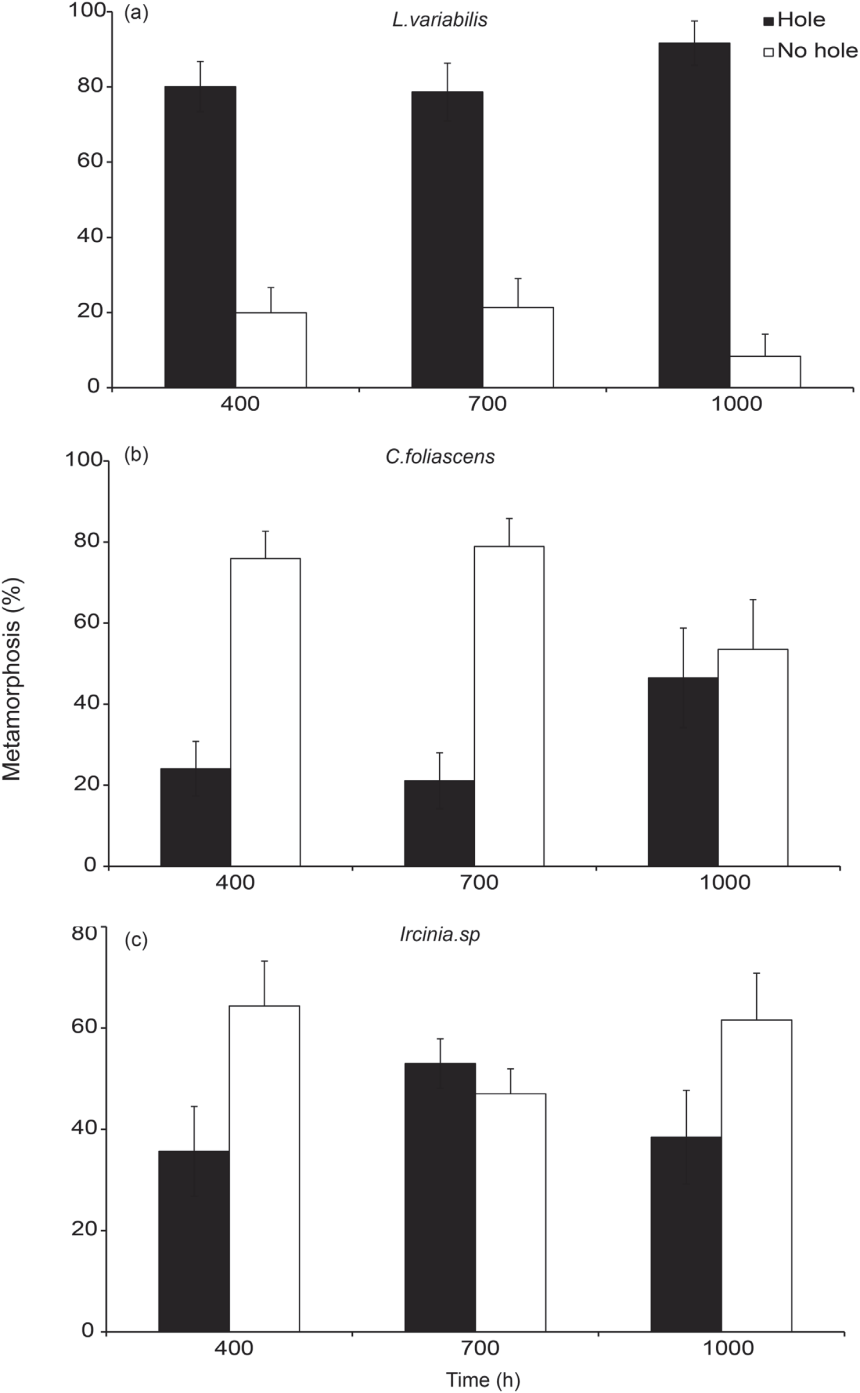
a-c. Larval metamorphosis(mean % + 1 SE) for the sponges, *Luffariella variabilis*, *Carteriospongia foliascens*, and *Ircinia* sp. that occurred in holes or no holes (flat inter-hole surfaces) for surface microtopography treatments of 400 μm, 700 μm and 1000 μm diameter holes. Settlement occurred within 3 days of spawning for all three species.

The outcome of whether settlement was a reflection of randomly selecting a hole or the flat surfaces in between holes varied among species (Fig. 5 a-b, Fig. 6 a-c). *A*. *millepora* showed clear patterns of selectivity for treatments of 400 μm and 700 μm holes with 100% of larval settlement occurring in holes for both these sized treatments (Fig. 5a—no inferential statistics computed). However, for 1000 μm treatments there was no significant difference between *A*. *millepora* settlement to holes or to flat surfaces (Fig. 5a, Binomial test p = 0.09). *C*. *crassa* larvae selectively settled in 400 μm holes (Fig. 5b, Binomial test 400; p < 0.01), but showed no pattern of selective settlement in treatments containing larger holes (700 μm and 1000 μm).

*L*. *variabilis* also engaged holes to undergo settlement irrespective of surface (size) treatment (Fig. 6a, Binomial Test 400 μm p < 0.01, 700 μm p < 0.01, 1000 p < 0.01). *C*. *foliascens* larvae showed a significant preference to settle on the flat surfaces of 400 and 700 μm treatments but no distinction was observed in 1000 μm treatments (Fig. 6b, Binomial Test: 400 & 700 μm, p < 0.01; 1000 p = 0.09). *Ircinia* sp. also showed significant biases to settle on flat (inter hole) surfaces in 400 μm and 700 μm treatments but not in 1000 μm treatments (Fig. 6c, Binomial Test: 400 p < 0.01; 700 μm p < 0.03; and 1000 μm p = 0.26).

## Discussion

The notable outcome of this study was that surface microtopography plays a substantial role in settlement for some larvae, in part supporting previous studies on corals and sponges that have established the importance of surface topography in larval recruitment [37–40]. While these studies demonstrated settlement to surface topography, the surface structures investigated were considerably larger than dimensions of larvae or propagules. The present study also showed larvae respond to surface structure, and our focus of matching larval sizes to surface microtopography reinforces the premise that larvae are more likely to settle to sizes that closely match their own larval dimensions. This is clearly demonstrated for *A*. *millepora* and *L*. *variabilis*, which showed a strong preference to settle in 400 μm holes, with this size hole closely matching the mean width of both species (434 μm—*A*. *millepora* and 391 μm—*L*. *variabilis*). Of interest, was the finding that *A*. *millepora* did not show any settlement to controls (no surface topography), and significantly less settlement to 700 μm and 1000 μm holes, these sizes being over two fold larger than larvae. This trend was also demonstrated for *L*. *variabilis*.

While larval settlement to surface microtopography that closely match larval sizes is largely unexplored for corals and sponges the importance of physical surface cues in larval settlement has been demonstrated for other taxa, particularly in the biofouling literature [34–36,44,47,53]. Here, there is compelling evidence that larval settlement is influenced according to the points to which they can adhere; larval settlement is increased with available attachment points and decreased when attachment points are reduced (i.e. Attachment Point Theory). This theory was developed from experiments to establish ways to impede larval settlement. However, using surfaces that match larval dimensions can also optimise settlement [44]. Attachment Point Theory also supports the findings of this study for *A*. *millepora*, *L*. *variabilis*, and to a lesser extent, *C*. *crassa*.

The results that both *A*. *millepora* and *L*. *variabilis* settle to surfaces without chemical-based-habitat cues are significant. There is a strong focus of work documenting the importance of the settlement of coral larvae to CCA [23,24]. *A*. *millepora* has shown repeated evidence of settling to CCA, although there are numerous examples that prevent generalisations of CCA being considered a universal coral larval settlement cue [31]. For example, when CCA is present, *A*. *millepora* larval settlement in laboratory conditions can vary between 30% to 80% and appears to be moderated by distinct species of CCA [24,54]. Moreover, some species of CCA elicit strong responses for some coral species while other CCA species show less convincing evidence as settlement cues [31]. Similarly, *L*. *variabilis* has shown increased larval settlement in the presence of conspecifics [17]. That both *L*. *variabilis* and *A*. *millepora* respond to chemical based settlement cues, but nevertheless showed a response to surface structure at a size closely resembling their larval dimensions, highlights the utility of physical cues and potentially a combined chemical-physical approach. Indeed, our results highlight the complexity and range of cues implicated in larval settlement. These first results highlight the need for larval settlement studies to include broader approaches that incorporate both physical and chemical cues. This is particularly noted for *A*. *millepora*, which in the present study showed no settlement to control surfaces, and in 100% of cases settled to the holes of 400 μm surfaces.

Settlement for two of the sponge species, *C*. *foliascens* and *Ircinia* sp. were in direct contrast to results for the two coral species, and the sponge *L*. *variabilis*. There was no evidence that settlement was optimised with surface microtopography for these two sponges, with settlement occurring irrespective of the surface treatment. While some sessile invertebrate species have strict requirements for larval settlement (e.g. death before dishonour- [55]) other taxa show less specificity and can settle without any apparent cue, particularly as larval competencies decrease (e.g. desperate larva hypothesis—[56]). Larvae that do not require specific settlement cues are arguably afforded advantages if habitat related cues are not encountered, although this strategy may also result in settlement to unfavourable habitats for these generalist species [57]. Achieving competence in the presence of cues (as for *A*. *millepora* and *L*. *variabilis)* may also translate to increased post settlement success by reducing risks associated with latent effects (i.e. reduced energy to grow-[58]). In this study *A*. *millepora* settlement occurred earlier in 400 μm holes in comparison to larger holes. Therefore if latent effects are considered for *A*. *millepora*, larvae encountering and responding to settlement cues early in the pre-settlement phase may also increase post settlement survival. This should be a focus of further research as the ability to enhance both settlement and post-settlement survival will positively impact on concepts such as the sustainable supply of corals for display and public education, as well as for reef restoration using larval as a seed source.

While this study focused on the role of physical cues it is also important to recognise the role of chemical cues in larval settlement. The importance of biofilms, for example, cannot be excluded. Biofilms are likely to have developed on the tiles during the period of the settlement assays (2–11 days) in the present study and potentially contributed to larval settlement. Despite this potential, the age of biofilm development also plays a role in levels of larval settlement, with naive biofilms being less important to larval settlement than mature biofilms [27,32,59]. There are also potential cues associated with plastics associated with settlement tiles used in the present study. Polycarbonates can emit specific compounds [60] and any resulting effects on larval settlement of species tested in this study remain undetermined.

In conclusion, this study has established that larval settlement can be driven by physical (microtopography) settlement cues. It also questions the sole reliance on chemical cues for larvae to settle. While the long term survival of recruits was not a focus of this study, future work incorporating both chemical and physical cues, coupled with data on post settlement survival will provide an increased understanding of the dynamics that drive larval settlement and recruitment.
